# The Break-Fast study protocol: a single arm pre-post study to measure the effect of a protein-rich breakfast on autophagic flux in fasting healthy individuals

**DOI:** 10.1186/s40795-022-00617-5

**Published:** 2022-11-01

**Authors:** Julien Bensalem, Leonie K. Heilbronn, Jemima R. Gore, Amy T. Hutchison, Timothy J. Sargeant, Célia Fourrier

**Affiliations:** 1 Lysosomal Health in Ageing, Hopwood Centre for Neurobiology, Lifelong Health Theme, South Australian Health and Medical Research Institute, Adelaide, South Australia Australia; 2 Adelaide Medical School, Faculty of Health and Medical Sciences, The University of Adelaide, Adelaide, South Australia Australia; 3 Obesity and Metabolism, Nutrition, Diabetes & Gut Health, Lifelong Health Theme, South Australian Health and Medical Research Institute, Adelaide, South Australia Australia

**Keywords:** Autophagy, Protein, Amino acids, Whey protein isolate, Nutrition, mTORC1, Macronutrient, LC3

## Abstract

**Background:**

Autophagy is a cellular process that cleanses cells and is particularly important during ageing. Autophagy has been extensively studied in vitro and in animal models and is known to be sensitive to nutrition. However, human data are limited because autophagic flux (autophagic degradative activity) has been challenging to measure in humans. This protocol paper describes the Break-Fast study, in which autophagic flux will be measured using a recently developed blood test, before and after ingestion of whey protein. This aims to determine whether an acute nutritional intervention can change autophagy in humans.

**Methods:**

A minimum of forty healthy participants (both male and female) aged 20–50 years, BMI 18.5–29.9 kg/m^2^ will be recruited into this single arm pre-post study. Participants will visit the clinic after an overnight fast for a first blood collection after which they will consume a whey protein-rich drink. A second blood collection will be performed 60 minutes after consumption of the drink. The primary outcome is the change in autophagic flux at 60 minutes post drink. Secondary outcomes include changes in blood glucose, autophagy-related proteins and mRNA, plasma hormones (e.g. insulin, C-peptide, adiponectin, GLP-1, GIP, ghrelin), cytokines, amino acids and lipids, protein synthesis, and correlation between molecular cell damage and autophagic flux.

**Discussion:**

This study will provide information about whether autophagy responds to nutrients in humans, and if nutritional strategies could be used to treat or prevent autophagy-related diseases such as Alzheimer’s disease or cancer.

**Trial registration:**

Australian New Zealand Clinical Trials Registry (ANZCTR), anzctr.org.au ACTRN12621001029886. Registered on 5 August 2021.

**Supplementary Information:**

The online version contains supplementary material available at 10.1186/s40795-022-00617-5.

## Background

Autophagy is an intracellular process that is critical for healthy cellular function and the prevention of age-related disease. Autophagy collects and degrades unwanted or damaged organelles and macromolecules, and replenishes macronutrients during nutritional stress. Preclinical research has shown that autophagy delays the onset of atherosclerosis and Alzheimer’s disease-related signs [1, 2]. Manipulating autophagy has therefore become an important field of research to prevent or delay age-related disease. Nutrition-mediated regulation of autophagy pathway activity is well documented, with many in vitro studies showing that nutrient restriction induces autophagy, notably through inhibition of the mechanistic target of rapamycin complex 1 (mTORC1) [3, 4]. In contrast, fewer studies have documented this response in animal models and even less in humans.

Whilst autophagy is the name of the pathway by which cargo are delivered to the lysosome, autophagic flux is the measure of the degradation activity of this pathway over a period of time. Measuring autophagic flux in humans is particularly challenging because the existing methods to measure autophagic flux require either genetic manipulation (e.g. a transgene coding for a fluorescent probe) [4–6], or the use of a lysosomal inhibitor such as chloroquine to block degradation of autophagic material by the lysosome [6]. Amounts of autophagy protein microtubule-associated proteins 1A/1B light chain 3B II (LC3-II), a marker of autophagic vesicles, can be measured to determine how much autophagic material should have been degraded during the lysosomal inhibition period in comparison to a non-treated paired sample. This method has been used to measure autophagic flux in human peripheral blood mononuclear cells (PBMCs) ex vivo [7–10]. However, maintaining the cells in artificial cell culture medium is unlikely to reflect autophagy in a physiologically relevant context, notably due to their nutrient-rich composition [11], known to alter autophagy [4].

To overcome this limitation, we recently innovated on this method by treating the whole blood sample with the lysosomal inhibitor chloroquine rather than PBMCs in artificial culture medium, therefore maintaining more physiologically relevant conditions [10, 12, 13]. With this method, a blood sample treated with chloroquine and an untreated paired control blood sample from the same person are incubated for 1 h and maintained at 37 °C. PBMCs are then isolated and autophagic flux is measured using LC3-II quantification by enzyme-linked immunosorbent assay (ELISA). This method is the most physiological measure of human autophagic flux to date [13] and allows researchers in the field to reliably measure the effect of pharmacological and lifestyle interventions on autophagy in humans.

Nutritional interventions could be powerful methods to change autophagy and improve health. These interventions are thought to work at least in part through deactivation of the autophagy repressor mTORC1, which in itself is activated by insulin and the branched-chain amino acid, leucine. However, the hypothesis that nutritional interventions can be used to modify autophagy is based on in vitro and animal studies, or on clinical studies which measured indirect or snapshot readouts of autophagy that correlate poorly with autophagic flux [14]. Our new method that measures autophagic flux while cells are in whole blood finally allows measurement of the effect of nutrition on autophagic flux in humans. We recently reported that mimicking a nutrient-rich environment by treating blood samples ex vivo with the amino acid L-leucine and with insulin, (known activators of mTORC1 [15, 16]) decreased autophagic flux [10]. However, this result is preliminary and the ability of a nutrient-rich environment to inhibit autophagic flux in humans must be confirmed in vivo. This is a crucial step towards the development of long-term nutritional strategies that change autophagy and subsequently improve health.

The aim of this study is to establish whether acute ingestion of whey protein inhibits autophagic flux in healthy people compared with a fasted state. Baseline autophagic flux, measured after an overnight fast, i.e., when autophagic flux is expected to be the highest, will be compared with autophagic flux after study participants consume a whey protein-rich drink, which is known to rapidly increase plasma amino acids and insulin [17, 18]. We will additionally measure change in blood glucose before and after the ingestion of the whey protein drink and explore whether protein intake changes plasma hormones, cytokines, amino acids and lipids, protein synthesis, and molecular cell damage and how these changes correlate with changes in autophagic flux.

## Methods and design

### Ethics and study registration

Ethics approval for this study was obtained from the University of Adelaide Human Research Ethics Committee (HREC) on 22 February 2021 (HREC reference number: H-2021-024_V6.0_18/07/2022). Any and all serious adverse events to occur considered to be related to the study are reported to the aforementioned HREC by the principal investigator. In the event of participants suffering harm due to the study, the study is covered by indemnity insurance.

The study was registered on anzctr.org.au with the following identifier: ACTRN12621001029886. The study is a single site study performed at the South Australian Health and Medical Research Institute (SAHMRI) in Adelaide, South Australia, Australia, by researchers from SAHMRI and the University of Adelaide.

### Study design

The study is a pre-post, single arm clinical study. Participants are their own control, and fasting status is considered the baseline measurement.

### Participants

Participants are recruited from the Adelaide metropolitan area (South Australia, Australia). The study is advertised through SAHMRI and The University of Adelaide networks as well as social media.

Participant inclusion and exclusion criteria for the study are listed in Table 1.

**Table 1:** Participant inclusion and exclusion criteria

Inclusion criteria	
Aged between 20 and 50 years of age	
BMI between 18.5 and 29.9 kg/m^2^	
Exclusion criteria	
Co-morbidities that are likely to change the activity of the lysosomal system, such as cancer, diabetes, cardiovascular disease/conditions (stroke, heart attack, high blood pressure), major psychiatric disorders (schizophrenia, addiction, eating disorders, major depressive disorder), neurological disorders, dementia.	
Any lifestyle (e.g. physical activity, dietary habits) and/or change in lifestyle deemed likely to affect the results by the study principal investigator.	
Taking any medications that might change autophagic activity; including, but not restricted to, medications that change body composition or metabolism (e.g. medications used to lower blood glucose, antidiabetic medications), anti-inflammatory medications, medications that change brain function (e.g. antidepressant medications, mood stabilisers, lithium), medications used in the treatment of cancer or cardiovascular disorders.	
Female participants taking hormonal contraceptive will not be excluded from the study, if they have a regular menstrual cycle, they will be asked to attend the study appointment between day 1 and day 10 of their cycle.	
Current alcohol and/or substance use disorder.	
Lactose intolerance (except if managed by medication e.g. Lacteeze).	
Vegan.	
Allergies preventing consumption of the study product.	
Current smoker.	
Pregnant or breastfeeding women or women planning a pregnancy.	
Women who have been through menopause.	
Anyone who is unable to comprehend the study protocol (i.e. due to English language or cognitive difficulties).	

### Screening

Interested participants are invited to contact the study team by email or by phone and are sent an online screening questionnaire to complete via REDCap (Research Electronic Data Capture) to determine initial eligibility. Participants are also given the opportunity to complete the screening questionnaire by phone or in person if they request it. The screening questionnaire gathers demographic, and health-related information, including medical and exercise history, to assess their eligibility for the study. Participants are also provided with a copy of the participant information sheet (Additional file 1) and allowed as much time as they require to decide whether they would like to participate.

Potentially eligible participants are invited to attend a subsequent study appointment at SAHMRI following a 12-hour fast. A member of the study staff explains about the concept of autophagy, the study objectives, risks and potential benefits of participation, and obtains consent from the participant (Additional file 2). Participants are free to withdraw from the project at any time.

If participants meet the eligibility criteria, they are invited to continue in the remainder of the study.

### Randomisation

No randomisation is required in this study as all participants undergo a fasted blood collection, and a second blood collection 60 minutes after consuming a drink containing 30 g of whey protein isolate. Participants act as their own control.

### Study intervention

Participants are asked to consume a standardised unflavoured whey protein isolate powder (30 g of powder: Energy 492 KJ, Protein 27.0 g, Fats 0.4 g, Carbohydrates 0.7 g) diluted in skim milk (250 mL: 350 KJ, Proteins 8.25 g, Fats 0.25 g, Carbohydrates 11.5 g) in less than 5 minutes. This protein-rich drink is prepared on site by study staff just before consumption by the participant. Consumption of a similar protein-rich drink is known to increase plasma amino acids and insulin rapidly after ingestion, with peak concentrations of plasma branched-chain amino acids, such as leucine, occurring after 90 minutes, and peak plasma insulin occurring after 60 minutes [17, 18].

### Study visit

Participants are asked to refrain from strenuous exercise and alcohol the day before the study visit. After an overnight fast (12 hours, with permission to drink water), participants attend the study visit at SAHMRI. Study visits are booked between 8:00–9:00 am at the participants convenience. Upon arrival, the study is explained to the participant in detail, and informed consent is obtained. Participants are required to give consent for collection and storage of blood samples (in the form of whole blood, serum, plasma, RNA and/or cell populations), consumption of a protein-rich drink, collection of de-identified clinical data (age, gender, fasting duration, self-report of medical history including family history of dementia), and routine clinical measurements (blood pressure, weight, height, waist and hip circumferences) performed at the study visit to determine eligibility.

Blood pressure is measured after a minimum 10-minute seated rest, and weight and height measurements obtained to assess body mass index and confirm eligibility. Waist and hip circumference are measured. All anthropometric measurements are collected using standardised procedures.

Following collection of anthropometric measurements, a fasting blood sample (20 mL) is collected. Immediately after the blood collection, participants are asked to consume the standardised whey protein drink in less than 5 minutes. After a resting period of 60 minutes post drink consumption, a second blood sample (30 mL) is collected. During the resting period, participants are permitted water (but no other food or drinks) and asked to remain sedentary.

**Fig. 1:**
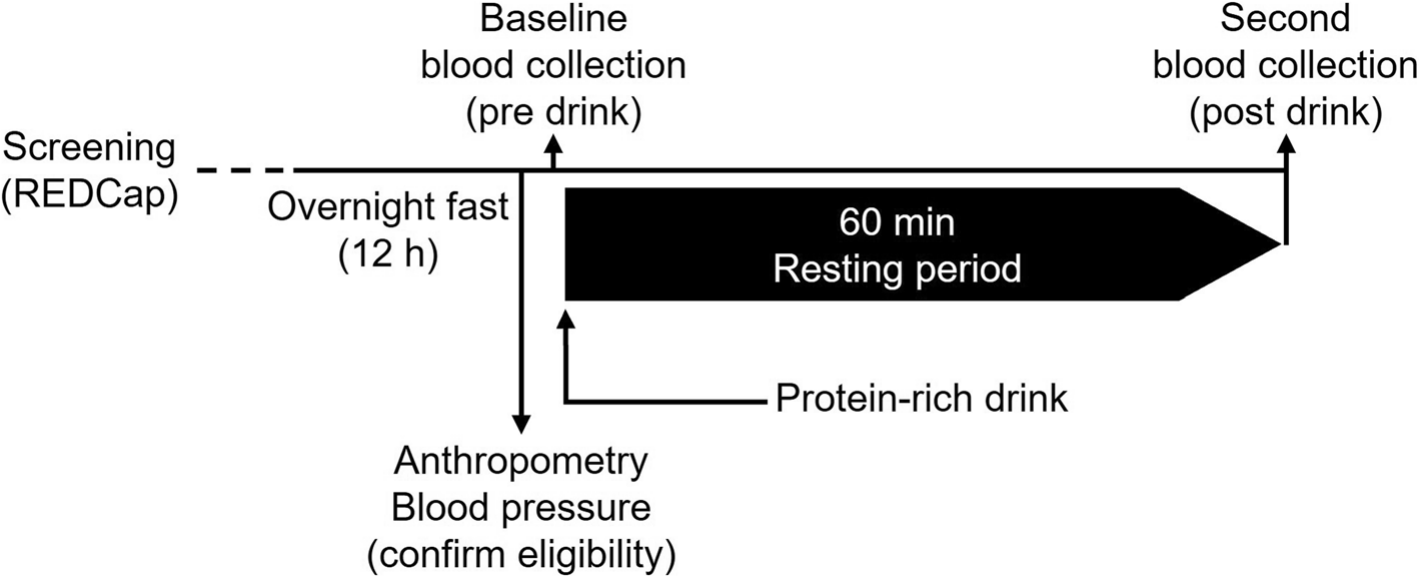
Experimental design of the Break-Fast study

The study flow chart is illustrated in Fig. 1.

**Fig. 2:**
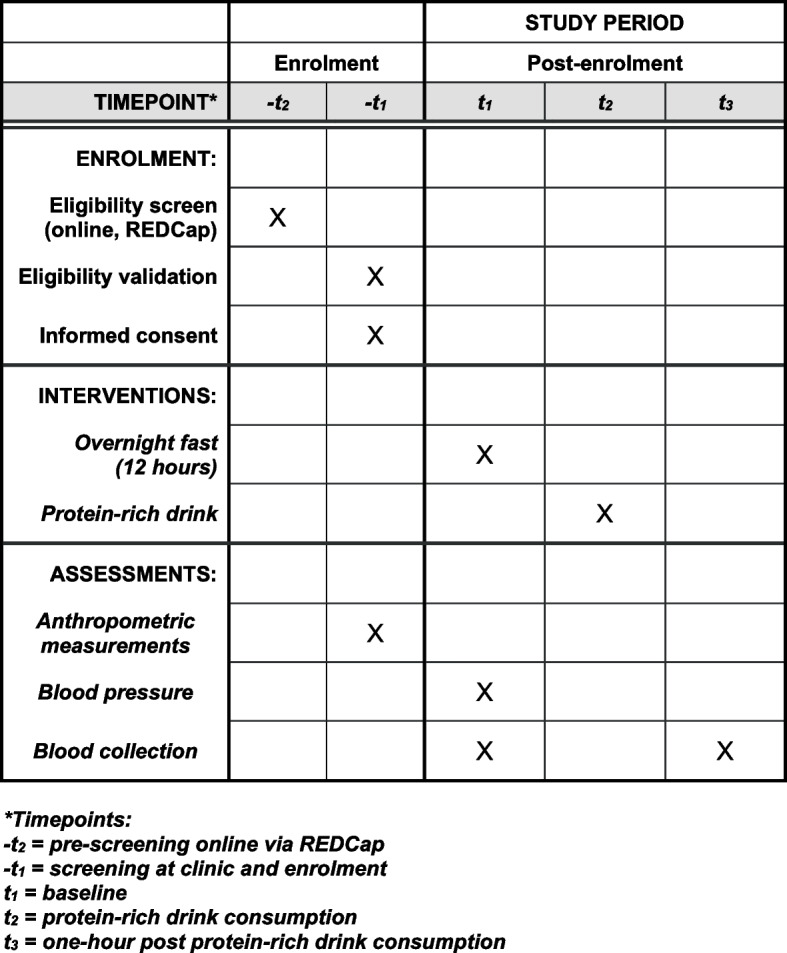
SPIRIT figure. Schedule of enrolment, interventions, and assessments

An additional SPIRIT (Standard Protocol Items: Recommendations for Interventional Trials) checklist and an additional figure have been provided for further details regarding the study of enrolment, intervention, and assessments for the current study (Additional file 3 and Fig. 2).

### Planned outcomes

#### Primary outcome measure

- ΔAutophagic flux (between before and after whey protein drink consumption)

#### Secondary outcome measure

- ΔBlood glucose concentration (between before and after whey protein drink consumption)

#### Exploratory outcome measure

- ΔAutophagy related proteins and mRNA levels (between before and after whey protein drink consumption)
- ΔPlasma hormones and cytokines levels (between before and after whey protein drink consumption)
- ΔPlasma amino acids levels (between before and after whey protein drink consumption)
- ΔPlasma lipids levels (between before and after whey protein drink consumption)
- ΔProtein synthesis (between before and after whey protein drink consumption)
- Correlation between molecular cell damage and autophagic flux
- Identification of molecules that correlate with autophagic flux
- Autophagic flux and inflammatory markers (protein and gene expression) in monocyte-derived microglia-like cells.

### Outcome measurements

#### Anthropometric data

Height is measured without shoes to the nearest 0.1 cm using a height measuring rod stadiometer. Weight is measured after voiding, with clothing to the nearest 0.1 kg using a calibrated scale. Waist circumference is measured at the mid-axillary line (halfway point between lowest rib and the top of iliac crest), and hip circumference is measured at the widest circumference of the buttocks using a metal measuring tape. Body mass index (BMI) is calculated as weight in kilograms divided by height in metres squared.

#### Blood pressure

Blood pressure is measured with the participant seated after 10 minutes of rest.

#### Autophagic flux measurement

Blood samples is processed immediately after collection for measurement of autophagic flux according to the method developed by our group [10]. Briefly, 6 mL of the blood samples is split into two tubes, one tube treated with, and the other without chloroquine (CQ) (which blocks lysosomal degradation of autophagic cargo LC3-II), and incubated for 1 h at 37 °C. PBMCs are then isolated from both tubes and stored frozen at − 80 °C before biochemical analysis of protein cargo accumulation LC3-II by ELISA, using a commercially available kit. Autophagic flux will be determined by the difference in the accumulation of LC3-II over time between blocked and unblocked samples (i.e., what should have been degraded during the one-hour incubation) using the equation ΔLC3-II = (LC3-II_+CQ_ – LC3-II_-CQ_)/hour.

#### Autophagy-related markers

Complementary autophagy-related markers will be measured in order to provide a better picture of the autophagic machinery and its status. Change in autophagy-related proteins and mRNA will be assessed by western blot and/or RT-qPCR (e.g., phospho-mTOR, phospho-S6 ribosomal protein, phospho-5′ adenosine monophosphate-activated protein kinase [AMPK], Sequestosome-1 [P62/SQSTM1], Lysosomal-associated membrane protein 1 [LAMP1]). Molecular cell damage (e.g., lipid oxidation, oxidative stress) will be analysed using commercially available assays. Protein synthesis will be measured in PBMCs as it is thought to be increased after protein-rich meal consumption. Furthermore, this has never been analysed in concomitance with autophagic flux which is thought to inversely correlate with protein synthesis. Biological material (e.g. plasma, leukocytes) isolated and stored during this project may be used to identify autophagy biomarkers via, but not limited to, proteomic, metabolomic and/or lipidomic analysis. In addition, autophagic flux and inflammatory markers (protein and gene expression) will be studied in monocyte-derived microglia-like cells obtain during this project (exploratory).

#### Plasma markers and amino acid concentrations

To assess the metabolic changes induced by the whey protein drink, plasma is snap frozen at − 80 °C for later assessment of blood glucose, insulin, blood lipids, free fatty acids, glucose regulating hormones (e.g., insulin, C-peptide, adiponectin), inflammatory cytokines (e.g., C-reactive protein [CRP]), and appetite-regulating hormones (e.g., Glucagon-like peptide-1 [GLP-1], Glucose-dependent insulinotropic polypeptide [GIP], ghrelin) using commercially available kits. Plasma amino acids will be analysed by mass spectrometry. These measures will allow us to directly assess the covariance between metabolic parameters and autophagic flux.

### Sample size calculations

Based on pilot data from experiments conducted on 10 individuals fasted for 12 hours, the standard deviation (SD) measured for autophagic flux was 0.3287 [10]. After an ex vivo nutrition-related intervention aimed at decreasing autophagic flux (similar to what is tested in vivo in this study) a 0.64-fold change was observed and the observed SD was 0.4185 [10]. Considering the SD observed, the SD(Δ) = 0.55 (calculated from the observed SD of the outcome and the within-subject correlation of the outcome), and *N* = 38 participants, this would provide 80% power to detect a medium effect size of 0.3, with an alpha risk of 0.05 (paired-t test, ‘before-after’). A minimum of forty individuals (both male and female) will be recruited for this study. No blinding will be implemented during this study as everyone’s samples will be paired (pre- post-intervention) for analysis.

### Statistical plan

The primary outcome of this interventional study with paired samples (pre- post-intervention) taken from each participant will be determining whether autophagic flux (ΔLC3-II) decreases in response to a protein-rich drink. After confirming the normality of this variable with the Shapiro-Wilk test, a paired sample t-test (or Wilcoxon signed rank test) will be used to test the mean score difference in ΔLC3-II before and after the protein-rich drink. The same method will be applied to the secondary outcomes. Correlations will be tested using simple linear regression. Effect of covariates such as baseline value, age, or sex, will be analysed at posteriori. Missing data will not be imputed. Demographic characteristics will be summarised using descriptive statistics.

### Data management

Upon screening, participants are assigned a unique study-specific identifier that is used on all data collection documentation after confirming their enrolment in the study. Personal information of participants is stored in a separate file from the main database in order to protect confidentiality. All paper-based documentation will be kept for 15 years by researchers in a secure and locked cabinet. All electronic data collection and documentation is password protected and can only be accessed by designated research team members.

Biospecimens are stored at SAHMRI in secured freezers, all of which are monitored by a central alert system 24 h/day 7 days a week.

No Data Monitoring Committee (DMC) will be required as this study does not involve significant safety concerns, risks or complexity.

The results of the study will be made publicly available via official publication and media release at the study’s conclusion.

## Discussion

The Break-Fast study will provide the first evidence of modulation of autophagic flux by nutrition in healthy individuals. Specifically, this study will determine if an acute intake of protein decreases autophagic flux as measured in blood. This study will indicate whether we can detect acute changes in autophagic flux in humans and therefore be able to measure the effect of future nutritional, lifestyle or pharmacological interventions aimed at changing autophagy to promote health.

## Trial status

This study was registered on the 5th of August 2021 at anzctr.gov.au under identifier ACTRN12621001029886. The first participant was enrolled on the 10th of March 2022. Recruitment was completed on the 2nd of September 2022. At the time of submission, all de-identified participants samples are stored and have not been processed for analysis yet. If any important changes to the study protocol become necessary, where appropriate, the principal investigator will notify the HREC, trial register and the present journal. The study is subject to audit at any time by the HREC.

## Supplementary Information

**Additional file 1.** Study information sheet. Detailed study information is provided to participants during screening, before the study visit, and before written consent form is obtained.

**Additional file 2.** Participant consent form. The written informed consent is obtained by the participants before starting the study.

**Additional file 3.** SPIRIT checklist. SPIRIT checklist listing all the items addressed in the current study protocol.

## Data Availability

Not applicable.

## Abbreviations

*AMPK*: 5′ adenosine monophosphate-activated protein kinase
*ANZCTR*: Australian New Zealand Clinical Trials Registry
*BMI*: Body mass index
*CQ*: Chloroquine
*CRP*: C-reactive protein
*ELISA*: Enzyme-linked immunosorbent assay
*GIP*: Glucose-dependent insulinotropic polypeptide
*GLP-1*: Glucagon-like peptide-1
*HREC*: Human Research Ethics Committee
*LAMP1*: Lysosomal-associated membrane protein 1
*LC3-II*: Microtubule-associated proteins 1A/1B light chain 3B II
*mTORC1*: Mechanistic target of rapamycin complex 1
*P62/SQSTM1*: Sequestosome-1
*PBMCs*: Peripheral blood mononuclear cells
*REDCap*: Research Electronic Data Capture
*SAHMRI*: South Australian Health and Medical Research Institute
*SD*: Standard deviation
*SPIRIT*: Standard Protocol Items: Recommendations for Interventional Trials
