# Supplementary material for: The Break-Fast study protocol: a single arm pre-post study to measure the effect of a protein-rich breakfast on autophagic flux in fasting healthy individuals
Source: BMC Nutr. 2022 Nov 1;8:120. doi: 10.1186/s40795-022-00617-5 (PMC9623954; doi:10.1186/s40795-022-00617-5)
Supplement: Supplementary file 1 — Additional file 1. Study information sheet. Detailed study information is provided to participants during screening, before the study visit, and before written consent form is obtained. [file 40795_2022_617_MOESM1_ESM.pdf]

PARTICIPANT INFORMATION SHEET

PROJECT TITLE:

Effect of a protein-rich Breakfast on autophagy in Fasting healthy people (Break-*Fast* study)

HUMAN RESEARCH ETHICS COMMITTEE APPROVAL NUMBER: H-2021-024
PRINCIPAL INVESTIGATOR: Dr Timothy Sargeant

Dear Participant,

You are invited to participate in the research project described below.

What is the project about?
Autophagy is a cellular process that cleanses your cells and is particularly important as we age. Fasting or caloric restriction have been at the core of strategies that seek to boost autophagy to prevent cell damage and ageing. Despite numerous media flooding the internet advocating the advantages of diets that boost autophagy, all our knowledge mostly comes from work done in the laboratory and in rodents, not humans.

To answer questions about whether nutritional strategies can change autophagy in humans scientifically, our research group has developed a first-ever test to measure autophagy in human blood.

This project aims to measure in autophagy in blood from fasted participants and how autophagy changes after consuming a protein-rich liquid meal. This will provide information about whether autophagy responds to nutrients in humans, and if and how nutritional strategies could be used to treat or prevent diseases known to be impacted by change in autophagy such as Alzheimer's disease or cancer.

Who is undertaking the project?
This project is being conducted by Dr Timothy Sargeant, Dr Julien Bensalem, Dr Célia Fourrier, Ms Kathryn Hattersley, Ms Leanne Hein, Ms Jemima Gore and Ms Daniela Calderisi (SAHMRI); and A/Prof Leonie Heilbronn, Dr Bo Liu, Dr Amy Hutchison and Ms Xiao Tong Tong (University of Adelaide). Additional personnel may join the research team at any time to assist with this project.

Why am I being invited to participate?
You are being invited to participate as you are aged between 20 and 50 years, with a stable weight and a BMI between 18.5 and 29.9 kg/m^2.

What am I being invited to do?
First, we will assess your eligibility to participate in the study with a screening questionnaire (questions regarding your diet, exercise and medical history). The completed questionnaire can be returned by email, or you will be offered the opportunity to complete the screening process over the phone or during a face-to-face appointment if this is your preference. If you are eligible for the study, and decide that you would like to take part, you will be invited to attend a study appointment at SAHMRI.

You will be asked to come into the SAHMRI research facility in the morning after a 12-hour overnight fast (water intake will be permitted). We will ask that you refrain from strenuous exercise and alcohol the day before the study visit. This visit will take approximately 1.5 hours. During this appointment, we will explain

the study in detail to ensure that you understand what the study is about and what will be done if you decide to take part, and to answer any questions you may have.

If you decide to be a participant, we will perform routine clinical checks (e.g. measure your height, weight, blood pressure, waist and hip circumference). A blood sample will be collected (up to 20 mL). You will then be given a milk shake to drink (30 grams of whey protein isolate powder diluted in one cup of skim milk) and asked to consume it within 5 minutes. You will be asked to sit for 60 minutes after you have finished the milk shake. You will be permitted water but no food or other drinks. After 60 minutes, a second blood sample will be collected (up to 30 mL). You will be offered a complimentary $20 gift voucher after the second blood collection.

Possible risks associated with those procedures are detailed in the section “Are there any risks associated with participating in this project?”.

How much time will my involvement in the project take?
The screening questionnaire may take 5-15 minutes to complete online. If you cannot complete the questionnaire online, then a phone or face-to-face appointment can be arranged. If you are eligible to participate in the study, you will be invited to attend an appointment at SAHMRI. You will have the study explained to you and you will have two blood collections done, one prior to and one after consuming a provided high-protein shake. This appointment will take approximately 1.5 hours.

Are there any risks associated with participating in this project?
Disclosing your information: whilst all the information you provide to us will remain confidential, it may be subject to discovery in court or legal proceedings. This is a rare occurrence, but we are obligated to inform you of this risk. We will not disclose this information, or any information you provide to us, unless required to by law.

Blood samples: this test involves inserting a small needle into a vein in your arm. Inserting the needle can be associated with a small amount of pain and although complications are rare, it may occasionally cause bruising, localised bleeding, faintness and, in rare cases, infection. If you have ever experienced any of these complications in the past, bring them to the attention of the person performing the blood collection. We will take a baseline blood sample (up to 20 mL) when you arrive. We will take a second blood sample (up to 30 mL) 60 minutes after you finish the milk shake.

What are the potential benefits of the research project?
This study is not directly assessing a treatment for a disease, and as such, you will not directly benefit from participating. This and future studies will inform us about whether using nutritional intervention targeting autophagy could potentially be useful to treat or prevent diseases that have been shown to relate to autophagy, such as cancer, Alzheimer’s or cardiovascular disease. To date, researchers do not know whether autophagy-related measures may be indicators of your health; therefore, you will not be provided with your individual results, nor will we provide any counselling or advice regarding your results. Some of the other outcomes we measure (such as fasting blood glucose levels) may be indicators of health. If these measures are outside of the normal ranges, you will be notified and provided with the relevant information to take to your GP for further evaluation.

Can I withdraw from the project?

I understand that I am free to withdraw from the project at any time. Should I consent to future use of any remaining samples, I understand that I am free to withdraw my consent for future analysis by

contacting the Principal Investigator at any time within the next fifteen years. My samples will be destroyed, and my data will be excluded from any analysis not already undertaken, and that this will not affect my relationship with the University now or in the future.

What will happen to my information?

Confidentiality and privacy: at screening, you will be allocated a study ID code, and all your information will be de-identified. All data collected will be stored on a password-protected database. While all efforts will be made to remove any information that might identify you, as the sample size is small, complete anonymity cannot be guaranteed. However, the utmost care will be taken to ensure that no personally identifying details are revealed.

Storage: hard copies of signed consent forms and study information will be stored securely in a locked cabinet on Level 6, SAHMRI, North terrace, and will be stored for 15 years. Your blood samples will be de-identified and stored for up to 15 years in freezers located on Level 6, SAHMRI, North terrace.

Publishing: data collected from this study will be published (as summary data only) in scientific journals and presented at academic conferences. These disclosures will not identify you by name. At the end of the study, you will receive a summary report about the results of the study. This report will not identify you or any other participant by name.

Sharing: blood samples and data collected during the study may be shared with other researchers, in Australia or overseas, with your consent. These samples and/or data will be identified by your study ID code only. After 15 years, samples will be disposed of in clinical waste bins and destroyed. Where your cultural sensitivities are relevant, specific agreements can be made with you and we will ensure that they are fulfilled when disposing of samples.

You will be asked whether you consent to be contacted for future research projects. We will only record your contact details on a password-protected file and contact you for future studies if you agree for us to do so.

Your information will only be used as described in this participant information sheet and it will only be disclosed according to the consent provided, except as required by law.

Who do I contact if I have questions about the project?

Should you have any questions or concerns before, during or after the study, please feel free to contact Dr Timothy Sargeant on 8128 4940 or by email tim.sargeant@sahmri.com; Dr Julien Bensalem by email julien.bensalem@sahmri.com; A/Prof Leonie Heilbronn on 8128 4838 or by email leonie.heilbronn@adelaide.edu.au; or Dr Célia Fourrier by email celia.fourrier@sahmri.com.

What if I have a complaint or any concerns?

The study has been approved by the Human Research Ethics Committee at the University of Adelaide (approval number H-2021-024). The research will be conducted according to the NHMRC National Statement on Ethical Conduct in Human Research 2007, (updated 2018). If you have questions or problems associated with the practical aspects of your participation in the project or wish to raise a concern or complaint about the project, then you should consult the Principal Investigator (Dr Timothy Sargeant). If you wish to speak with an independent person regarding concerns or a complaint, the University's policy on research involving human participants, or your rights as a participant, please contact the Human Research Ethics Committee's Secretariat on:

Phone: +61 8 8313 6028

Email: hrec@adelaide.edu.au

Post: Level 4, Rundle Mall Plaza, 50 Rundle Mall, ADELAIDE SA 5000

Any complaint or concern will be treated in confidence and fully investigated. You will be informed of the outcome.

If I want to participate, what do I do?

In the first instance, contact the research team by email (autophagy@sahmri.com). We will send you a screening questionnaire for you to complete and return by email to determine your eligibility for the study. If you wish to do the screening process by phone or in person rather than by email, we can arrange a screening interview with you. If you would prefer to contact us by phone first, please call the Principal Investigator (Dr Timothy Sargeant; 08 8128 4940) and leave a message with your name and phone number. A member of the study team will call you back to discuss the study with you and answer any questions you may have. If you are eligible to participate in the study, we will ask you to either return the signed consent form by email prior to the study appointment or to sign it on the day of your study visit.

Yours sincerely,

The Study Team

Dr Timothy Sargeant, Dr Julien Bensalem, A/Prof Leonie Heilbronn, Dr Célia Fourrier, Ms Kathryn Hattersley, Ms Leanne Hein, Dr Bo Liu, Dr Amy Hutchison, Ms Xiao Tong Teong, Jemima Gore and Daniela Calderisi.
