# Supplementary material for: The Break-Fast study protocol: a single arm pre-post study to measure the effect of a protein-rich breakfast on autophagic flux in fasting healthy individuals
Source: BMC Nutr. 2022 Nov 1;8:120. doi: 10.1186/s40795-022-00617-5 (PMC9623954; doi:10.1186/s40795-022-00617-5)
Supplement: Supplementary file 2 — Additional file 2. Participant consent form. The written informed consent is obtained by the participants before starting the study. [file 40795_2022_617_MOESM2_ESM.pdf]

Human Research Ethics Committee (HREC)

CONSENT FORM

1. I have read the attached Participant Information Sheet and agree to take part in the following research project:

**Title:**	**The Break-Fast study: Effect of a protein-rich Breakfast on autophagy in Fasting healthy people**
**Ethics Approval Number:**	**H-2021-024**

2. I have had the project, so far as it affects me, and the potential risks and burdens fully explained to my satisfaction by the research worker. I have had the opportunity to ask any questions I may have about the project and my participation. My consent is given freely.

3. I have been given the opportunity to have a member of my family or a friend present while the project was explained to me.

4. Although I understand the purpose of the research project, it has also been explained that my involvement may not be of any benefit to me.

5. I agree to participate in the activities outlined in the Participant Information Sheet (i.e. two blood collections; one prior to and one after consuming a provided drink containing 30 grams of whey protein).

6. I understand that I am free to withdraw from the project at any time. Should I consent to future use of any remaining samples, I understand that I am free to withdraw my consent for future analysis by contacting the principal researcher at any time within the next fifteen years. My samples will be destroyed, and my data will be excluded from any analysis not already undertaken, and that this will not affect my relationship with the University now or in the future.

7. I have been informed that the information gained in the project may be published in a journal article, student's thesis, news article, conference presentations, website or report.

8. I have been informed that in the published materials I will not be identified, and my personal results will not be divulged.

9. I agree to my information being used for future research purposes as follows:
   Research undertaken by these same researcher(s) Yes ☐ No ☐
   Research undertaken by any researcher(s) Yes ☐ No ☐

10. I hereby provide 'extended' consent for the use of my data or samples in future research projects that are:
    - (i) an extension of, or closely related to, the original project: Yes ☐ No ☐

- (ii) in the same general area of research: Yes ☐ No ☐

11. I understand my information will only be disclosed according to the consent provided, except where disclosure is required by law.

12. I am aware that I should keep a copy of this Consent Form, when completed, and the attached Participant Information Sheet.

Participant to complete:

Full Name: ___________________________________ Signature: _____________________________

Date: __ __/ __ __ / __ __ __ __

Researcher/Witness to complete: [Not Applicable for electronically returned consent forms.]

I have described the nature of the research to ____

(print name of participant)

and in my opinion she/he understood the explanation.

Signature: _____________________________

Full Name: ____________________________

Position: ______________________________

Date: __ __/ __ __/ __ __ __ __
